# The cytoskeletal control of B cell receptor and integrin signaling in normal B cells and chronic lymphocytic leukemia

**DOI:** 10.1002/1873-3468.70045

**Published:** 2025-04-17

**Authors:** Abhishek Pethe, Tanja Nicole Hartmann

**Affiliations:** ^1^ Department of Medicine I, Medical Centre‐University of Freiburg, Faculty of Medicine University of Freiburg Germany

**Keywords:** actin, BCR, CD49d, chronic lymphocytic leukemia, cytoskeleton, integrin, signaling

## Abstract

B cells migrate within lymphoid organs during maturation and activation, processes orchestrated by the interplay between B cell receptor (BCR) signaling and microenvironmental cues. Integrins act as mechanoreceptors, linking BCR activation to cytoskeletal remodeling, facilitating immune synapse formation, antigen recognition, and extraction. BCR activation models describe receptor clustering and mechanical changes within the antigen–BCR complex. Upon activation, immune synapses form, enabling antigen extraction and downstream signaling. Integrins stabilize these synapses, amplify BCR signaling, and modulate BCR positioning *via* actin reorganization. In chronic lymphocytic leukemia (CLL), aberrant BCR signaling and integrins are major players in leukemic cell homing, prognosis, and therapy resistance. In this review, we summarize the current understanding of the interplay of BCR mechanics and B cell localization, with a particular focus on communication between BCR signaling and integrin‐mediated processes *via* actin dynamics. We give insights into normal B cell biology and then outline aspects typical to CLL.

## Graphical abstract

In lymphoid organs, antigen recognition and B cell receptor signaling rely on integrins and the cytoskeleton. Integrins act as mechanoreceptors, couple B cell receptor activation to cytoskeletal remodeling, and support immune synapse formation as well as antigen extraction. This review outlines the interplay between these components in normal B cells and its relevance and alterations in chronic lymphocytic leukemia.

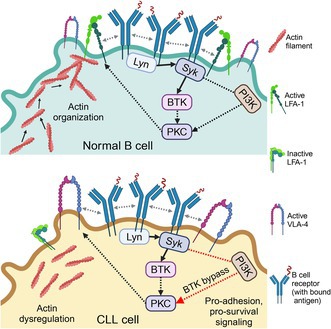

## Abbreviations

**APC**, antigen‐presenting cell

**BCR**, B cell receptor

**BTK**, Bruton tyrosine kinase

**CCL21**, C‐C motif chemokine ligand 21

**CCR7**, C‐C motif chemokine receptor 7

**CLL**, chronic lymphocytic leukemia

**cSMAC**, central supramolecular activation complex

**CXCL12**, C‐X‐C motif chemokine ligand 12

**CXCR4**, C‐X‐C motif chemokine receptor 4

**ERK1/2**, extracellular signal‐regulated kinases 1/2

**HS1**, hematopoietic‐specific protein 1

**ICAM‐1**, intercellular adhesion molecule‐1

**Ig**, immunoglobulin

**IGHD**, immunoglobulin heavy constant delta

**IGHJ**, immunoglobulin heavy‐chain, joining region

**IGHV**, immunoglobulin heavy‐chain variable region

**IGKV**, immunoglobulin kappa variable cluster

**IGLV**, immunoglobulin lambda variable cluster

**ITAM**, immunoreceptor tyrosine‐based activation motifs

***ITGB2***, integrin subunit beta 2

**LAD‐III**, leukocyte adhesion deficiency‐III

**LFA‐1**, lymphocyte function‐associated antigen 1

**M‐CLL**, IGHV‐mutated chronic lymphocytic leukemia

**NHL**, non‐Hodgkin lymphoma

**NOTCH1**, neurogenic locus notch homolog protein 1

**PI3K**, phosphoinositide‐3‐kinase

**PKC**, protein kinase C

**pSMAC**, peripheral supramolecular activation complex

**RIAM**, Rap1‐GTP‐interacting adaptor molecule

**ROR1**, receptor tyrosine kinase like orphan receptor 1

**THATCH**, Talin/HIP1R/Sla2p actin‐tethering C‐terminal homology domain

**TP53**, tumor protein P53

**U‐CLL**, IGHV‐unmutated chronic lymphocytic leukemia

**VCAM‐1**, vascular cell adhesion molecule 1

**VH‐CDR3**, third complementarity‐determining region of immunoglobulin variable genes for the heavy chain

**VLA‐4**, very late antigen‐4

**WASP**, Wiskott‐Aldrich syndrome protein

**ZAP70**, zeta chain of T cell receptor associated protein kinase 70

## Pre‐synopsis

B cells are a major component of the adaptive immune system and the humoral immune response. In order to fight pathogens, they can differentiate into antibody‐secreting plasma cells or memory B cells upon activation. The activation of a mature B cell begins when a B cell encounters an antigen *via* its B cell receptor (BCR). The range of antigens recognized by B cells is highly diverse, and BCRs themselves are remarkably diverse and versatile. Since the interaction between the antigen and BCR dictates the fate of a B cell, BCR signaling is critical in both normal function and malignancy. Antigens and supportive signals for B cell activation such as T cell help are not randomly distributed in the human body but are localized to highly specific tissue sites within lymphoid organs. Therefore, effective BCR signaling relies on the proper localization of the B cells juxtaposed to the supportive cues.

During their maturation, activation, and differentiation, B cells migrate through the lymphoid organs in a very orderly sequence, receiving signals tailored to each stage of activation. This migration is orchestrated through adhesion, mediated by heterodimeric transmembrane receptor proteins known as integrins and cytoskeletal changes within the cell. Therefore, BCR signaling, along with integrin‐mediated signaling, governs B cell activation, survival, and positioning for subsequent steps. Errors at any point in this journey can lead to malignant transformation, often resulting in B cell malignancies that retain features of their original subset.

It is not yet fully understood how B cell localization and BCR signaling are intertwined. Recent findings suggest that these two distinct types of signals are not merely integrated but more closely linked than previously thought. Mechanosensitive components of BCR signaling may play a role in how cells sense their location. At the same time, the location may have a direct impact on BCR signaling.

In this review, we summarize the current understanding of the connecting mechanics between BCR‐ and integrin‐induced modulations in the actin cytoskeleton and signaling. We will address both normal B cell biology and malignant B cell processes, with an emphasis on chronic lymphocytic leukemia (CLL).

## Concepts of BCR activation

From the earliest steps on, B cell development follows a tightly regulated sequence of stages occurring in distinct anatomical locations. B cell development begins in primary lymphoid organs, that is, human fetal liver and bone marrow, followed by functional maturation in secondary lymphoid organs such as spleen and lymph nodes. In secondary lymphoid organs, B cells must be activated by an antigen to continue their differentiation toward their ultimate plasma or memory B cell fate (for review, see [1]). This activation of B cells happens when they encounter an antigen presented by antigen‐presenting cells (APCs) such as macrophages or, for example, follicular dendritic cells, which are specialized stromal cells in the spleen and lymph nodes. Antigen encounter is facilitated by the formation of an immune synapse between the two cell types, as described in detail in the following chapters.

The BCR complex consists of surface immunoglobulins (Ig) that are noncovalently linked with the transmembrane proteins CD79A and CD79B. Their intracellular domains containing immunoreceptor tyrosine‐based activation motifs (ITAMs) transduce signaling to intracellular effectors [2]. Upon BCR activation, the ITAMs are reorganized, allowing Src family kinases such as Lyn to be recruited to the BCR complex [3]. Lyn phosphorylates the tyrosine residues within the ITAMs, creating docking sites for other signaling proteins, such as Syk [4]. Activated Syk amplifies the signaling cascade by further phosphorylating downstream substrates, which helps recruit additional kinases such as Bruton's tyrosine kinase (BTK) [5]. The kinase recruitment dynamic is dependent on the actin cytoskeleton, further explained in later chapters.

A remarkably diverse range of molecules can engage the BCR, including, but not limited to, small, soluble antigens such as hen egg lysozyme, ovalbumin, and bovine serum albumin, as well as larger insoluble viral and bacterial fragments and anti‐immunoglobulin antibodies [6, 7]. In fact, the various BCRs can recognize almost any ligand of any origin. This is due to the dual expression of both IgM and IgD as the components of mature, naïve BCRs, allowing for a broader antigen recognition due to differences in hinge region flexibilities of both the Ig (reviewed by [8]). IgD BCRs can substitute IgM BCR under certain circumstances [9] for each other, with IgD being able to induce a stronger and prolonged phosphorylation of downstream protein tyrosine kinases than IgM [10]. However, upon recognition of an autoantigen, the B cells downregulate IgM on their surface, which is more sensitive to autoantigens, thus preventing the launch of an autoimmune attack [11, 12]. In lieu of this, probably in an effort to prevent autoimmunity, the threshold of mechanical forces necessary to trigger IgM BCRs is higher than other BCR isotypes [13]. BCR triggering by an antigen causes BCR downstream signaling and remarkably, this signaling is influenced by the characteristics of the respective antigen, such as its affinity or valency. In addition, the mechanical characteristics of the APC, such as their membrane stiffness, impact BCR signaling. An increase in membrane stiffness of follicular dendritic cells was shown to promote a more stringent affinity discrimination by the B cell, a process strongly dependent on the actin cytoskeleton [14]. When the actin cytoskeleton was disrupted, the associated decrease in membrane stiffness led to different membrane components being extracted and internalized by the B cell, possibly also leading to different B cell responses depending on what parts of the antigen‐presentation complex are being extracted [15]. This incredible complexity of B cells might be one of the reasons why antigen recognition mechanisms in B cells are still incompletely understood.

Several models have been proposed to explain the initial events of BCR stimulation by antigen. The classical and earliest model suggests that the BCR exists in monomers that are cross‐linked and cluster together upon antigen binding [16]. In line, multivalent antigens are more powerful in triggering B cell activation. Förster resonance energy transfer [17, 18] and direct stochastic optical reconstruction microscopy [19, 20] studies demonstrated the spatial organization of BCRs into large clusters upon multivalent antigen binding. An alternative model, the dissociation–activation model, suggests that pre‐formed auto‐inhibited BCR oligomers dissociate upon antigen binding [21], allowing exposure of signaling sites, thereby relieving the autoinhibition. This model could be valid for large as well as small antigens. The antigen footprint model, also known as the kinetic aggregation model, provides another perspective by suggesting steric aspects. In this model, the antigen needs to occupy a certain footprint around the BCR Fab binding site, which subsequently shifts the balance of the intracellular kinases and phosphatases from a state of dynamic equilibrium toward activation [22]. Each of these models has strengths and limitations, and no consensus has yet been reached. It is possible that different B cell subsets employ distinct mechanisms of activation, and different models can therefore co‐exist. This could also shed light on certain BCR characteristics observed in malignancies, such as stereotyped CLL.

## The structure and activation of integrins

The adhesion receptor family of integrins is well known for their key role in extravasation and cell migration of B cells. Less known but equally important is their role in stabilizing immune synapses and contributing to BCR signaling. Due to the nature of integrins being mechanoreceptors, and since they link to the actin cytoskeleton, it is evident that integrins modulate BCR clustering processes, the actin retrograde flow (for details, see following chapters) and antigen affinity modulation. Integrins thereby prevent autoimmunity, are involved in BCR‐autonomous and antigen‐triggered signaling, and influence which model of antigen recognition might be used by the various B cell subsets. Here, we outline the structural basics of integrins and detail the molecular cues involved in the interplay of integrins with the actin cytoskeleton and the BCR in B cells.

All integrins are composed of two membrane glycoproteins, an alpha and beta subunit, that are non‐covalently associated [23]. The extracellular protein domains of both these glycoproteins are in the form of elongated stalks with a globular ligand‐binding head. In humans, alternative splicing leads to different variants of α and β subunits being produced (18 α and 8 β), allowing for 24 different heterodimerization combinations [23]. While the cytoplasmic tails of the different α subunits of the various heterodimers differ significantly from each other (except for a conserved motif that is necessary for the α‐β subunit interaction), the β‐subunits show a noticeable homology, especially the conserved NxxY sequence, which is involved in indirectly tethering the integrin to the cellular cytoskeleton [24]. At least 11 heterodimer combinations are expressed on B cells [23], with the two integrins leukocyte function antigen‐1 (LFA‐1, the CD11a/CD18 heterodimer) and very late antigen‐4 (VLA‐4, the CD49d/CD29 heterodimer) being the two most important.

**Fig. 1:**
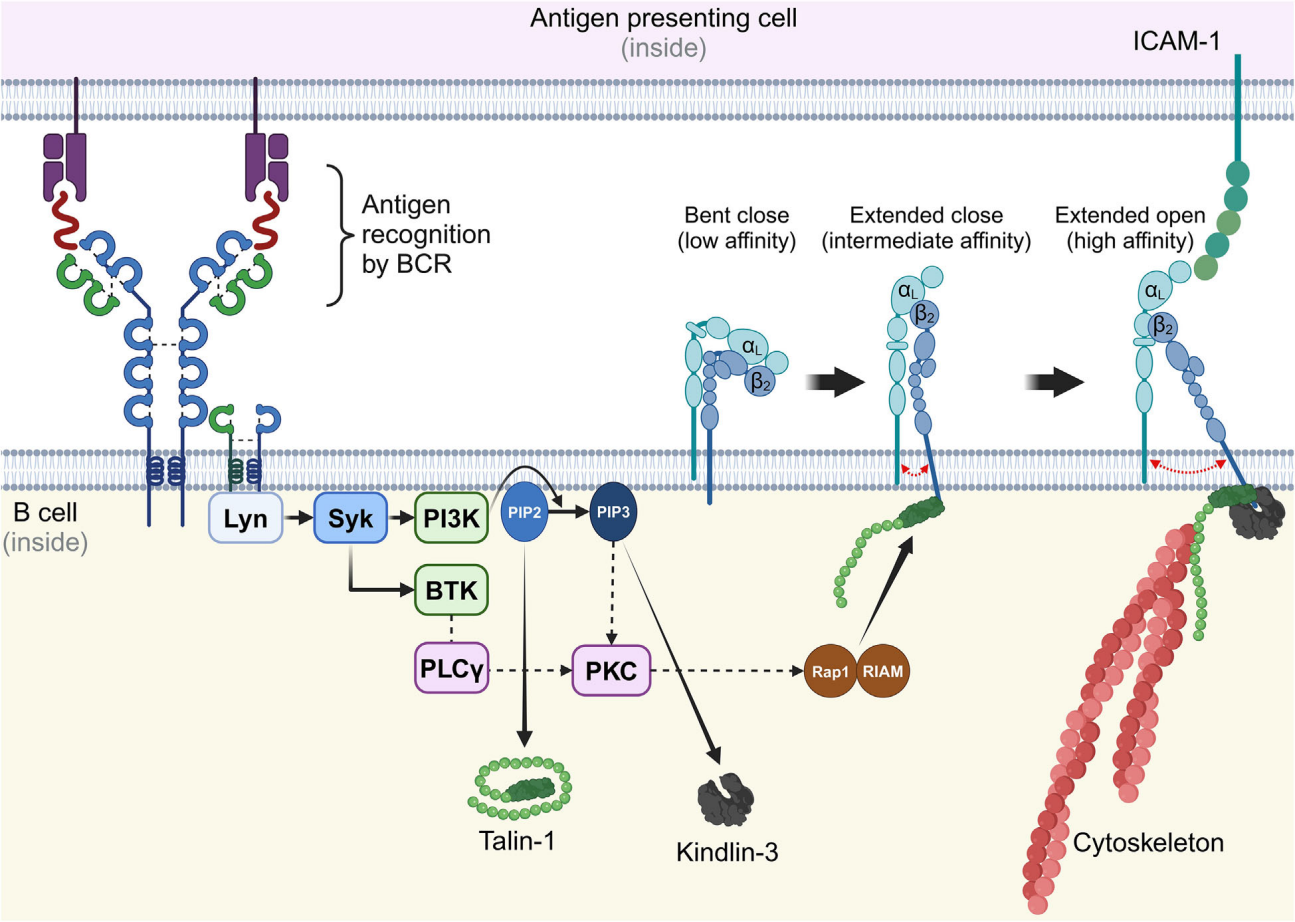
Inside‐out activation of the integrin LFA‐1, in normal B cells, by co‐operative binding of talin‐1 and kindlin‐3. Antigen recognition by the BCR leads to the activation of the membrane resident kinase Lyn and, subsequently, Syk. Syk activates BTK, activating downstream signaling pathways such as PKC *via* PLCγ signaling, which is well established in B cells. Based on studies in leukocytes other than B cells, we presume that subsequently PKC signaling activates Rap1, which, in association with its adaptor protein RIAM, as well as PIP2, promotes the binding of talin‐1 to the β subunit of LFA‐1, leading to an ‘extended close’ conformation with intermediate affinity. Syk also activates PI3K, which promotes PIP2 conversion to PIP3, leading to kindlin‐3 recruitment, which, along with the physical forces exerted by talin‐1‐bound Actin cytoskeleton, drives LFA‐1 into a high‐affinity ‘extended open’ conformation, enabling binding to ICAM‐1 on antigen‐presenting cells.

To reach the highest adhesive strength, integrins need to be activated, which involves conformational changes of the integrin structure. Most of the current knowledge of this process comes from research on LFA‐1 [25, 26]. Briefly, in the inactive form, LFA‐1 adopts a bent‐closed conformation, with the ligand‐binding site hidden. In an extended‐closed conformation, the extracellular domains swing away from the cell membrane but remain associated at the transmembrane domain regions, leading to a low ligand‐binding affinity. Subsequently, the associated cytoskeleton pulls the cytoplasmic tails of the alpha and beta subunits apart [27], leading to an extended‐open conformation and complete exposure of the ligand‐binding site (high affinity conformation) (Fig. 1). VLA‐4 displays more possible conformations and is more rapid in its alteration, which is partly due to its structural difference: in contrast to LFA‐1, the CD49d subunit of VLA‐4 lacks a regulatory I‐domain, enabling faster ligand‐binding kinetics. With at least six structures supporting low, intermediate, and high affinity binding, this integrin is unique in enabling not only firm cell adhesion but also rolling of cells on the substrates [28].

A rapid cascade of intracellular signaling events, the so‐called inside‐out signaling, triggers the integrin conformational changes (Fig. 1). It begins with the activation of a cell surface receptor such as a cytokine, chemokine, or antigen receptor [29], involves several kinases and G proteins, and eventually leads to the conformational changes of the integrin and its lateral mobility on the cell membrane [27]. Integrin clusters and adhesive spots are created. Alterations of integrin conformation and clustering (i.e., distribution on the cell membrane) can occur individually or simultaneously depending on the nature of the stimulation. Core components of the cascade include PI3K and PKC [29].

The actin cytoskeleton controls how integrins move on the lipids of the plasma membrane, thereby rearranging the positioning of these proteins along with the BCR aggregates and associated kinases. Bringing these proteins closer together or separating them determines BCR downstream signaling characteristics. In resting B cells, BCRs are located outside of lipid rafts, while in activated B cells, BCR microsignalosomes are trapped inside the lipid rafts. Only activated integrins can associate to lipid rafts [30]. This is likely due to the spatial separation of their intracellular subunits upon inside‐out activation, which allows attachment to the cytoskeleton for transport into the microsignalosome‐containing lipid rafts. This process further strengthens the adhesive properties of the B cell.

Inside‐out activation of integrins starts with an external trigger, and the kinetics of this process are dependent on the nature of this trigger, the localization of the cell, and the mechanical forces. Chemokine‐induced integrin activation is usually required for extravasation under the shear stress of the blood flow and therefore very rapid, taking place in fractions of a second. The mechanical force of the shear stress contributes to the kinetics and the activation strength [31]. In contrast, BCR‐induced integrin activation takes place more slowly but is more sustained (reviewed in [32]). This reflects the nature of the microenvironment, that is, the low to minimal shear stress within lymph nodes during antigen recognition. Here, the mechanical force is the stiffness of the APC [15]. The ability of B cells to differentiate surface stiffness due to integrin activation is especially important considering that follicular dendritic cells are usually stiff cells that allow for efficient antigen extraction by B cells, compared to softer cells such as dendritic cells.

## Integrin adaptors and cytoskeletal linkage

Upon full activation and ligand binding, integrin adaptors, such as talin and kindlin, stabilize the beta subunit of integrins and adhesion complex components, such as focal adhesion kinase and integrin‐linked kinase [33]. Together, these focal adhesions bridge the integrins and the cytoskeleton, allowing the propagation of chemical and mechanical signals that control cellular function and generate pulling forces inside the cell [33].

The talin family of proteins consists of two isotypes, talin‐1 and talin‐2, with talin‐1 being the linker molecule between integrins and the cytoskeleton. Upon binding to the integrin, talin‐1 leads to a change in the tilt angle between the integrin subunits, which causes them to be pulled apart, which is required for the ‘on’ state of the integrin. At the same time, talin‐1 behaves like a spring and unfolds to reveal multiple binding sites for actin and other focal adhesion complex components (Fig. 1) [34].

The kindlin family consists of three members, kindlin‐1, kindlin‐2, and kindlin‐3, with kindlin‐3 (gene name: *FERMT3*) being expressed exclusively in the hematopoietic system. Mutated or lost *Fermt3* leads to the severe condition of leukocyte adhesion deficiency‐III (LAD‐III), characterized by a lack of integrin activity in various immune cells, for example, platelets [35, 36, 37, 38]. Little is known about the role of kindlin‐3 in B cells. In mice, B cell‐specific loss of kindlin‐3 resulted in an expansion of follicular B cells with a loss of marginal zone B cells [39]. Chemokine and BCR‐induced inside‐out signaling of both LFA‐1 and VLA‐4 was impaired upon kindlin‐3 loss.

Talin‐1 and kindlin display a high sequence homology. Their F3 subdomain binds to the NxxY sequence of the integrin beta subunit. Talin‐1 interacts with the GTPase Rap1 and its corresponding adaptor protein RIAM, which exposes the β‐subunit binding site of talin‐1, allowing it to directly interact with the integrin and tether to the plasma membrane (Fig. 1) [40]. Evidence suggests that this Rap1‐RIAM‐talin‐1 interaction is necessary only for LFA‐1 activation, but not for VLA‐4 activation [41]. The actin‐tethering role of the adaptors is performed by the F0 domain in kindlin [42] and by multiple actin‐binding sites in the F2/F3 domain, the central region, and the long spring‐like C‐terminal domain of talin‐1 [43, 44, 45]. Extensive biophysical modeling and nuclear magnetic resonance studies have shown that both talin‐1 and kindlin are required for complete activation of integrin. Mutations in the genes of either protein abrogate the capacity of integrin activation. The mechanisms of integrin activation due to the conformational change induced by the binding of kindlin and talin are presumably the same across cell types, including B cells.

The C‐terminal domain of talin contains the talin/HIP1R/Sla2p actin‐tethering C‐terminal homology domain (THATCH domain), which coordinates talin dimerization and direct cytoskeletal F‐actin binding. The dimerization of talin seems to be a necessity for the talin–actin linkage [46]. The contraction of F‐actin filaments exerts a mechanical force on the integrin–talin complex and presumably exposes pockets in the rod‐domain of talin, which allows for the binding of F‐actin‐binding protein vinculin, further stabilizing the integrin–talin–actin complex [47]. Notably, the talin–integrin bond has been described as a so‐called slip bond under force. The bond is too weak to sustain the forces that act on talin or on integrin–ligand interactions [48, 49]. However, the cooperation of kindlin to form a ternary kindlin–talin–integrin complex significantly stabilizes the bond, converting it into an ‘ideal bond’—a force‐independent catch bond, withstanding more than four times the force of the slip bond [50].

## Immune synapse formation

**Fig. 2:**
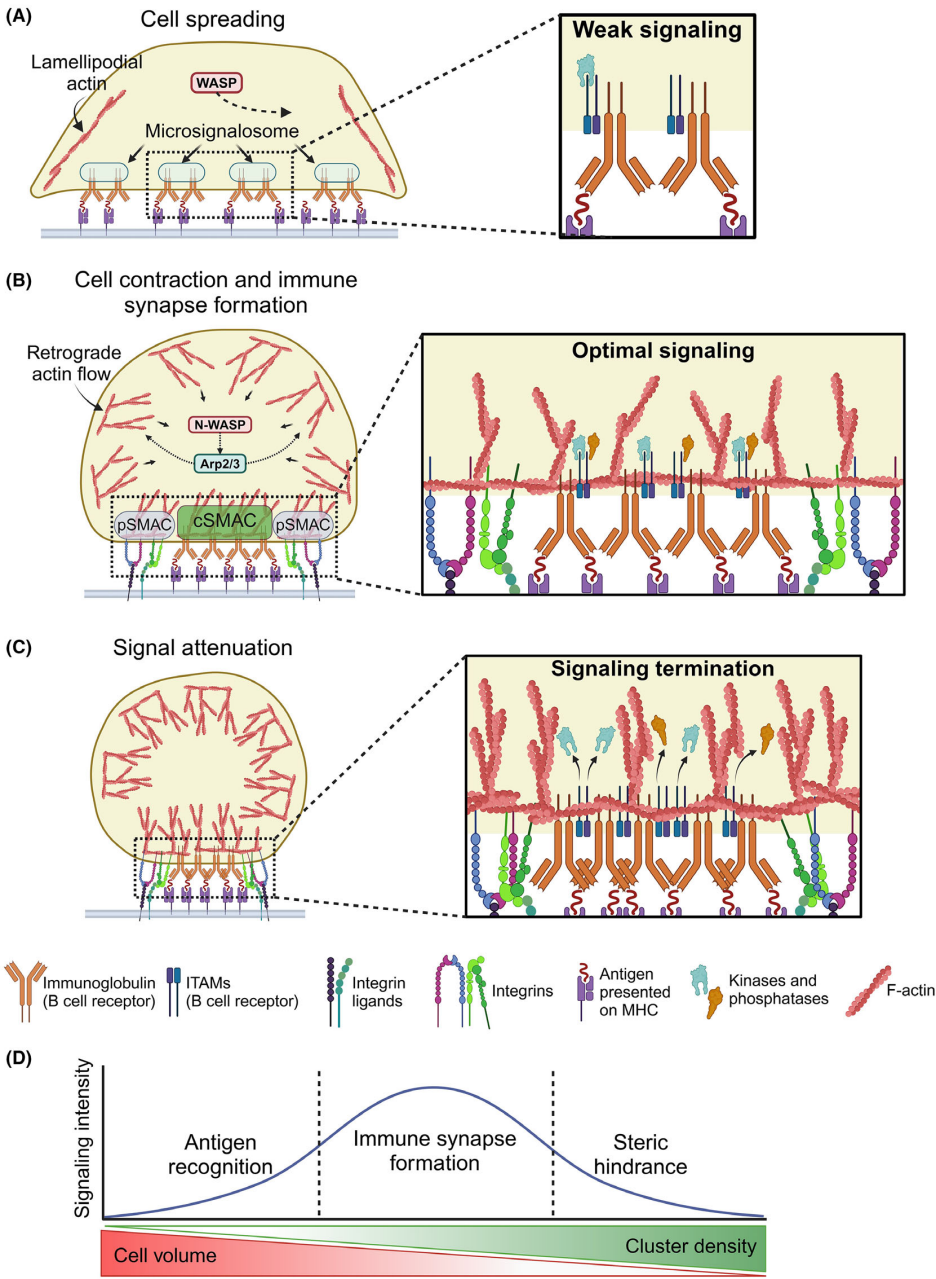
Regulation of immune synapse formation and B cell receptor signaling by Actin dynamics in normal B cells. (A) Following antigen recognition, WASP‐dependent F‐Actin polymerization promotes B cell spreading, allowing the BCR to bind more antigen and form microsignalosomes with weak signaling. (B) In the second phase of optimal BCR signaling, N‐WASP and Arp2/3 drive a retrograde Actin polymerization that leads to B cell contraction. This pulls microsignalosomes together into a central cluster, tethered to the Actin cytoskeleton, called the cSMAC. This proximity activates integrins to form the pSMAC. (C) As the B cell continues contracting, steric hindrance pushes BCR‐associated kinases and phosphatases out, attenuating BCR signaling. (D) Kinetics of normal BCR signaling intensity upon antigen recognition in relation to cell volume and BCR cluster density.

During antigen recognition of surface‐bound antigens, the B cell and the APC physically interact *via* an immune synapse. Thereby, the cytoskeleton is dynamically restructured in two phases: the spreading and the contraction phase (Fig. 2). B cell spreading enables the continuous collection of antigen and is driven by a lamellipodia‐like area containing branched actin (Fig. 2A), the distal supramolecular activation cluster (dSMAC). Lyn and BTK, as well as actin reorganization, are required for B cell spreading [51]. Loss of the actin nucleation protein Wiskott–Aldrich syndrome protein (WASP) leads to a significant reduction of B cell spreading. During the second phase, the contraction phase, all bound antigen is collected into a central supramolecular activation cluster (cSMAC), which is stabilized by an integrin‐ and filamentous (F)‐actin‐containing peripheral domain (pSMAC) (Fig. 2B). As the cell continues contracting, the density of the BCR clusters keeps on increasing, which leads to an inhibition of BCR signaling due to the dissociation of BCR‐associated kinases and phosphatases, and has been hypothesized to be due to molecular crowding at the clusters (Fig. 2C) [52]. Thus, upon antigen recognition in normal B cells, the cell volume decreases, the BCR cluster density increases, leading to an increase in BCR signaling intensity with an optimum in an immune synapse, and a subsequent decrease due to steric hindrance (Fig. 2D).

Even before or at the earliest point of spreading, BCR‐antigen microclusters, also termed microsignalosomes, are formed. This formation leads to a transient activation of Rap GTPase and subsequently the actin‐regulatory protein cofilin [53], which causes de‐polymerization of the existing cytoskeleton [54]. The disassembly of the actin cytoskeleton makes the B cell more flexible, promoting B cell spreading and enhancing the mobility of the BCR microclusters on the cell membrane. The extent to which the B cell is able to spread is dependent on the initial antigen affinity and density on the presenting surface [18]. Through the spreading process, the B cell develops more and more signaling‐active microsignalosomes. Upon reaching the minimum threshold of microsignalosome BCR signaling (determined by the intensity of intracellular BCR signaling *via* kinases and phosphatases), actin repolymerization by WASP and neural (N)‐WASP allows for further stabilization of the accumulated BCRs [55, 56]. The nature of the BCR, whether IgM or IgD, affects microsignalosome formation. The clustering of one BCR isotype functions independently of the other, implying that each isotype microsignalosome can initiate BCR signaling by itself [20]. IgD BCRs reside in lipid rafts and are associated with the co‐receptors and co‐activators CD19 [57], CXCR4, and CD22 [58] even prior to activation. IgM BCRs reside in nonlipid raft domains and only associate with these receptors postactivation and clustering [59].

In the case of soluble antigen recognition, the treadmilling movement of the re‐polymerizing actin drives the surface BCRs to one pole of the B cell and forms the so‐called ‘BCR cap’ [60]. In the case of membrane‐bound antigen recognition, the repolymerizing actin forms a ring‐like structure that drives the movement of the microsignalosomes toward the center of the cell, ultimately leading to the immune synapse [61, 62]. In the surrounding pSMAC, ongoing actin polymerization continues to gather new membrane‐bound antigen into what is now a matured immune synapse, forming a contractile F‐actin ring‐like structure that also consists of nonmuscle myosin II [63]. In either case, the clustering causes the amplification of downstream BCR signaling pathways [52]. Intracellularly, these microclusters are enriched in tyrosine‐phosphorylated proteins, building the base for the recruitment of signaling kinases such as Lyn and Syk [64].

The signals of weak antigens or low concentrations of antigens can be amplified by integrins. This process has been dissected in depth in mouse B cells [65]. It requires integrin‐linked actin to be rearranged into concentric, ring‐like structures composed of smaller arcs of F‐actin. These structures are created by mDia1, a formin family protein. Myosin IIA facilitates the contraction of these actin rings, which mechanically drag the antigen–BCR complexes to the center of the immune synapses to form the cSMAC. The mechanical stabilization of the immune synapse by these adhesive actions stabilizes and strengthens the BCR–antigen interaction to promote the formation of strong and mature immune synapses.

## Antigen extraction

Once a mature immune synapse has formed, the B cell extracts membrane‐bound antigens primarily through mechanical force [15]. This extraction involves either the physical rupture of the antigen from its tether or membrane deformation to facilitate antigen detachment. High‐affinity antigen–BCR complexes are usually larger than low‐affinity antigen–BCR complexes. They form large clusters enriched in actin and myosin IIa and resist a higher degree of mechanical stress. Lysosomes migrate toward the immune synapse site, supporting the antigen extraction process [66]. Afterwards, the antigens are internalized *via* clathrin‐mediated endocytosis and translocated from the plasma membrane into intracellular endosomal compartments. In this process, the actin cytoskeleton plays an important role. In the case of an APC‐B cell immune synapse, antigens are processed and presented at major histocompatibility complex II for CD4 T cell recognition [67]. Triggering of the BCR and subsequent actin de‐polymerization contribute to loosening the cortical actin cytoskeleton, thereby allowing the initiation of this process [15]. It is further promoted by Lyn, which phosphorylates the clathrin heavy chain. Clathrin‐coated pits are assembled, which are coupled to the actin cytoskeleton [68, 69]. BTK activates WASP and Vav, which are recruited to the surface‐bound BCR and to the internalized BCR‐antigen complex‐containing vesicles [70].

This coordinated rearrangement of the actin cytoskeleton, along with the support of integrins and integrin adaptor proteins, enables immune cells to effectively interact with their environment, facilitating B cell adhesion, migration, and signaling, while BCR clustering further refines cellular activation and response. Together, these processes ensure proper immune cell activation and localization, which are crucial for immune system function. A disruption of these dynamics is seen in B cell malignancies such as CLL, aspects of which are discussed below in detail.

## Chronic lymphocytic leukemia

Chronic lymphocytic leukemia (CLL) is a slow‐progressing non‐Hodgkin lymphoma (NHL) with an approximate yearly incidence of five cases per 100 000 persons in Europe. Biologically, CLL is a disease of mature, functionally incompetent B cells that harbor tissue‐specific characteristics. Proliferation takes place exclusively in lymphoid organs and is driven by an interaction of the leukemic cells with the microenvironment (as reviewed in [71]). This is supported by cues from T cells, stromal cells, and myeloid cells and is also driven by the BCR characteristics of the CLL cells.

CLL displays a high grade of heterogeneity. Some patients remain without the need for therapy for many years, while others present with an aggressive form that requires immediate therapy and/or progress to an aggressive lymphoma, referred to as Richter syndrome [72]. Prognostic factors such as the mutational status of the immunoglobulin heavy variable region (IGHV) genes reflect the biology of the B cell in the absence of treatment (reviewed in [73]). Prognostic factors can be intrinsic alterations such as genetic or genomic modifications of the leukemic cells, or markers of extrinsic interactions of the leukemic cells with the tumor microenvironment such as surface receptors (reviewed e.g., in: [74]), [75], with the BCR playing a dominant role [71].

Compared to normal B cells, CLL cells exhibit distinct BCR characteristics that underlie their malignant behavior [76]. Most CLL cells express both IgM as well as IgD BCR isotypes, with minimal evidence of mutual desensitization [77]. Prior incubation with anti‐IgM antibodies can enhance IgD‐induced calcium responses in some cases [77]. The chemotactic response of CLL cells differs depending on BCR activation, with anti‐IgM stimulation promoting CCR7‐directed migration within the lymphoid microenvironment [77]. Notably, the membrane levels and spatial organization of IgM, but not IgD, are associated with proliferative features, and only IgM BCRs mediate autonomous signaling [78]. The cytoskeletal protein hematopoietic‐specific protein 1 (HS1) is known as a critical component of CLL pathophysiology, with cytoskeletal remodeling representing a critical early event following BCR engagement, discussed also in later chapters [79, 80]. An association of robust HS1 activation and F‐actin polymerization is particularly observed upon anti‐IgD stimulation [81].

In the scope of this review, we will concentrate on BCR aspects that are relevant to the interaction of CLL cells with the microenvironment, which require cytoskeleton‐dependent processes and integration of very different external signals in these processes.

## The peculiarities of the BCR in CLL

Key aspects of the BCR in CLL are the IGHV status, the existence of quasi‐identical (also known as stereotyped) BCRs, and BCR‐autonomous signaling.

Somatic hypermutation is the most critical mechanism underlying the diversification of immunoglobulin genes. Hypermutations in the *IGHV* gene region occur in the germinal centers upon B cell activation and mirror the grade of antigen experience of a B cell. Therefore, the IGHV mutation status of CLL cells (with a 2% cut‐off) reflects their differentiation history and distinguishes two biologically distinct leukemic subtypes. IGHV‐unmutated CLL (U‐CLL) might originate from B cells that have undergone no or only little somatic hypermutation in germinal centers. They are more responsive to antigen, display stronger BCR signaling, and exhibit less anergy. U‐CLL BCRs tend to have a higher affinity for antigens, including autoantigens and microbial antigens. IGHV‐mutated CLL (M‐CLL) cells typically exhibit weak BCR signaling and a more anergic phenotype, characterized by reduced surface IgM expression, impaired calcium mobilization, and attenuated downstream signaling cascades. The biological differences manifest in strong differences in clinical outcomes, with U‐CLL having a poorer outcome than M‐CLL [82, 83]. Although these differences of U‐CLL versus M‐CLL have extensively been explored, the spatial BCR distribution at steady state and the earliest events upon activation, e.g., BCR clustering, are not fully resolved. Shorer Arbel *et al*. [84] demonstrated that the BCRs in unstimulated U‐CLL cells are preclustered along with a basal CD79a and BTK phosphorylation. U‐CLL BCRs polarized into large microclusters that formed patches and caps, while M‐CLL BCRs were more uniformly distributed across the cell membrane. The polarized BCR clusters co‐localized with the phosphorylated CD79a in U‐CLL, suggesting a constitutive cell‐intrinsic BCR signaling in this subgroup.

Besides the heterogeneity regarding somatic hypermutation, CLL displays another level of complexity, namely a skewed *IGHV* gene usage. About 30–40% of all human CLL cases present with highly conserved, and sometimes identical, variable heavy complementarity‐determining region 3 (VH‐CDR3) sequences. Such distinctly functionally and genetically similar BCRs are termed to be ‘stereotyped’ [85]. This implies that the antigen binding sites are very similar or quasi‐identical, pointing to shared antigenic drivers in the CLL evolution. CLL patients displaying the same stereotyped BCRs often share clinical features and courses [86]. This might be linked to the topography and responsiveness of the specific BCRs. The clustering of identical BCR molecules through self‐association, called homotypic aggregation, is driven by the nature of the complementarity‐determining region, which defines the antigen recognition, and therefore is evidently different in the various subsets.

The best characterized subsets are [87]:

Subset #1: This subset represents almost 2–3% of all CLL cases and is defined by a usage of clan I IGHVs/IGHD6‐19/IGHJ4/IGKV1(D)‐39, with mostly unmutated *IGHV* genes [86, 88]. The BCRs of these tumor cells are functional and likely interact with autoantigens, including oxidation‐related epitopes, calreticulin, which regulates cell adhesiveness [89], and cytoskeletal proteins like vimentin. Patients with this subset harbor an aggressive disease course.

Subset #2: In this subset, IGHV3‐21/IGLV3‐21 BCRs form strong homotypic interactions, which can drive tonic signaling in the absence of external ligands. The BCRs can react with autoantigens and the major actin‐regulating protein cofilin‐1. The *IGHV3‐21* genes in this subset exhibit variable levels of somatic hypermutation, with ~ 60% classified as M‐CLL [86, 90, 91]. Subset # 2 cases are associated with a dismal prognosis, comparable to that of patients with TP53 aberrations, independent of the IGHV status. Particularly aggressive is the subset in case of the additional R110 mutation, a somatic hypermutation‐driven glycine‐to‐arginine substitution in the lambda chain, enabling cell‐autonomous signaling [92].

Subset #4: This subset is defined by IGHV4‐34/IGKV2‐30 BCRs, with a remarkably indolent clinical course and mutated *IGHV* genes. Patients belonging to this subset are diagnosed at a relatively younger age compared to other CLL stereotyped subsets [85]. A possible explanation for the indolent disease course could be the functional anergy of the BCRs in this subset, leading to constitutively active ERK1/2 and a failure to release intracellular calcium [93, 94].

Subset #8: This subset, along with subset #4, expresses IgG isotype BCRs and is defined by IGHV4‐39/IGKV1(D)‐39 BCRs. These patients have functional BCRs and an aggressive disease. They are more prone to Richter's transformation due to the presence of trisomy 12 and NOTCH1 mutations [95], with CLL cells that express particular high CD49d levels [96]. The BCRs from this subset have a broad antigen recognition and bind both microbial and autoantigens, such as vimentin. BCR signaling is even more robust than that of subset #1 and #2, which might be the reason for disease aggressiveness [97].

Another hallmark feature of CLL cells is their capacity for cell‐autonomous BCR signaling without antigen stimulation, which is enabled by peculiarities in the complementarity‐determining region and an internal epitope of the BCR [98]. The distinct BCR stereotypes exhibit specific homotypic BCR aggregations that initiate qualitatively different cell‐autonomous signaling [99]. The nature of the signaling is associated with the clinical CLL course, with stronger affinities and longer half‐lives in indolent cases and weaker, short‐lived contacts in aggressive cases. Cell‐autonomous signaling capacity of CLL was also observed in the TCL1 transgenic mouse model for CLL, in which it was complementary to antigen‐responsiveness [100].

## Deregulation of actin in CLL

CLL cells have long been recognized to exhibit deregulations in the cytoskeleton as well as lipid composition, making these cells fragile. As early as 1982, it was reported that the actin and tubulin content of CLL cells is approximately one‐third lower than in normal lymphocytes [101]. Similarly, reduced levels of F‐actin, total actin, and vimentin were observed in lymphocytes from CLL patients compared to healthy donors [102]. These deficiencies lead to a fragile cytoskeleton, evident in the characteristic smudge cells observed in the blood smear of CLL patients. The actin defects are closely associated with the reduced migratory capacity of CLL cells [103, 104]. Early studies also revealed significant interpatient heterogeneity in cytoskeletal characteristics, particularly in vimentin expression and in the phenomenon known as CLL capping [105]. CLL cells are softer than normal B cells. Within CLL subgroups, IGHV‐unmutated cells are even softer than IGHV‐mutated cells, indicating that reduced cortical stiffness is associated with disease progression [106]. In addition, there is a positive correlation of the expression of the adaptor protein Tandem pleckstrin homology domain protein 2 and ZAP70, a marker of poor prognosis in CLL. This promotes CLL cell adhesion and plays a (PI3K‐induced) role in regulating the actin cytoskeleton, along with utrophin and syntrophin, contributing to rapid disease progression [107, 108]. Treatment with the BTK inhibitor ibrutinib was shown to restore the cytoskeletal stiffness to levels comparable to healthy B cells [106]. Although increased cytoskeletal softness may enhance CLL cell migration, its impact on BCR signaling remains unclear.

Cortactin, a homolog of HS1, has been identified as a key component at the intersection of BCR and actin signaling. The p80/85 splice variant of cortactin is overexpressed in high‐risk CLL subgroups and acts as a substrate for Src family kinases such as Lyn [109]. Cortactin orchestrates the recruitment and activation of the Arp2/3 complex to actin, promoting F‐actin polymerization and branching [110]. In patients with high cortactin expression, c‐Cbl, an E3 ubiquitin ligase, is constitutively associated with cortactin, unlike normal B cells, and correlates with poorer prognosis [111]. Activation of ROR1 by Wnt5a promotes binding of ROR1 to cortactin, triggering actin reorganization essential for chemokine‐directed CLL cell migration. The BCR proximal kinases Lyn and BTK thereby link BCR signaling and the cytoskeleton by phosphorylating cortactin. Consistent with this, ibrutinib treatment has been shown to reduce cortactin phosphorylation and partially restore actin defects in CLL cells [112].

## Integrins in CLL

Integrin expression and function are dysregulated at multiple levels in CLL. In normal B cells, both VLA‐4 and LFA‐1 are robustly expressed, with LFA‐1 known to play a critical role in immune synapse formation with follicular dendritic cells. In CLL, however, integrin‐related mechanisms display significant heterogeneity and complexity, likely reflecting the diverse BCR characteristics of tumor cells. Not only are the integrins altered, but also their linkage to the actin cytoskeleton and downstream signaling including defects in small GTPases such as Rap1 [113] and Rac [114].

### LFA‐1 (CD11a/CD18)

Early studies identified markedly decreased expression of LFA‐1 as a CLL characteristic [115, 116], with an inverse correlation between LFA‐1 expression and the degree of leukocytosis [115]. This is consistent with the key role of LFA‐1 in lymph node homing and retention of immune cells. It is reasonable to assume that the lowered LFA‐1 expression of CLL cells facilitates their egress from lymphoid organs into the circulation, possibly as a response to overcrowding in lymphoid tissues. Subsequent studies revisited the reduced lymph node homing of CLL cells attributed to LFA‐1 defects [104, 113]. CLL cells harboring low LFA‐1 are unable to transmigrate across the endothelium expressing VCAM‐1, ICAM‐1, and chemokines, which would be needed for effective lymph node homing [104]. The impact of impaired LFA‐1 on BCR activation in CLL remains poorly understood. However, it is plausible that severely reduced ICAM‐1‐dependent actin processes and immune synapse formation are disrupted. Rap1, a critical mediator of cytoskeletal rearrangements downstream of BCR signaling, is activated by the BCR, regulates the BCR repertoire, and contributes to the amplification of ERK signaling, a hallmark of anergy [117, 118, 119]. In CLL, defects in Rap1 and integrin recycling have been observed [113], warranting further investigation into their consequences for BCR signaling.

### VLA‐4 (CD49d/CD29)

**Fig. 3:**
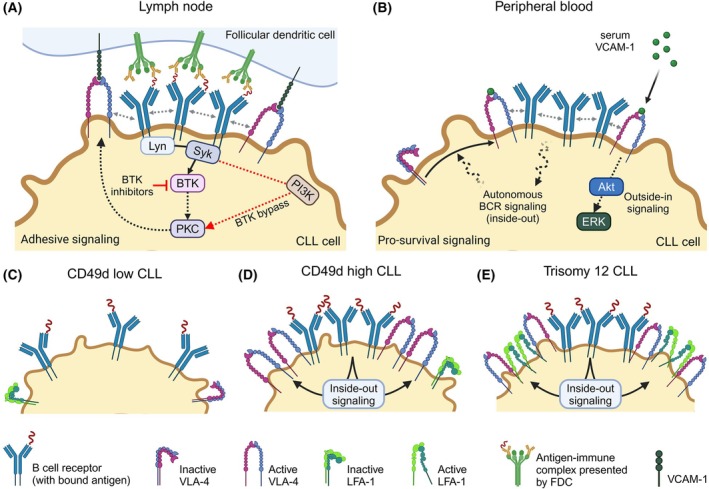
B cell receptor signaling and integrin crosstalk in CLL. (A) In lymphoid organs, antigen recognition can trigger BCR downstream signaling that activates the VLA‐4 integrin, contributing to tissue retention. While BTK inhibitors interrupt BCR signaling, CLL cells can bypass BTK in regard to VLA‐4 activation through pathways such as PI3K, conferring resistance to BTK therapy. (B) In the peripheral blood, BCR‐autonomous signaling can trigger VLA‐4 activation, along with enhanced binding to serum VCAM‐1, initiating pro‐survival, outside‐in activation of Akt and ERK. (C) CLL cells expressing low CD49d (and thus, VLA‐4), have presumably fewer BCR clusters due to limited BCR‐integrin interaction. (D) The VLA‐4 of CD49d^hi^ CLL cells is activated by inside‐out signaling upon antigen recognition by the BCR, without relevant contribution of LFA‐1, which is lowly expressed on these cells. (E) CLL cells with trisomy 12 express high levels of VLA‐4 as well as LFA‐1, both of which are activated by inside‐out BCR signaling.

VLA‐4 expression varies due to differences in transcription of the alpha4 subunit CD49d [104]. CD49d is expressed in approximately 40% of cases and serves as an independent negative prognostic factor in CLL, predicting unfavorable disease progression [120, 121]. Patients are categorized as CD49d‐low (< 30% CD49d‐positive CLL cells, Fig. 3C) or CD49d‐high (≥ 30%, Fig. 3D) based on flow cytometry analysis of peripheral blood CLL cells [120, 121, 122]. About a fifth of CLL patients exhibit a bimodal CD49d expression pattern, characterized by coexisting CD49d‐low and CD49d‐high subpopulations [123]. CD49d‐high and bimodal patients have worse overall survival and progression‐free survival (PFS) under chemoimmunotherapy and BTK inhibitor treatment compared to CD49d‐low patients [123, 124, 125]. In CD49d bimodal patients with multiple lines of therapy, the CD49d‐positive cell population increases over time, suggesting a resistant nature of this CLL cell population [123].

The biological role of the CD49d heterodimer VLA‐4 as a homing receptor, enabling CLL cells to anchor within protective niches in lymphoid organs and receive survival and proliferation signals from stromal, T, and myeloid cells, has long been established [104, 126]. However, recent findings reveal an additional, previously overlooked component: the BCR‐VLA‐4 interaction. In fact, VLA‐4 activation in CLL is not only triggered by chemokine cues but also through antigen‐dependent as well as autonomous BCR signaling [124, 127].

The antigen‐dependent VLA‐4 activation is particularly relevant for CLL cells residing in lymphoid organs and can occur independently of BTK *via* the PI3K axis [124]. As a result, CD49d‐positive CLL cells possess an additional mechanism to retain in lymphoid organs and to interact with antigen‐presenting cells such as follicular dendritic cells, even under BTK inhibition (Fig. 3A,D). In line with this, CD49d expression has been identified as a marker of shorter progression‐free survival in patients treated with BTK inhibitors like ibrutinib or acalabrutinib [124, 125, 128].

In the circulating CLL pool of CD49d expressing patient subgroups, serum VCAM‐1 can bind to activated VLA‐4, triggering outside‐in signaling cascades involving ERK activation and actin polymerization, contributing to the survival of this pool during the blood journey (Fig. 3B) [129]. Circulating CLL cells can also express pre‐activated VLA‐4 as a consequence of BCR‐autonomous triggering, further enhancing the binding of the serum VCAM‐1 and the subsequent survival effects [127]. In this blood compartment and in the context of BCR‐autonomous signaling, in contrast to the situation of antigen stimulation within the lymph node microenvironment, ibrutinib is capable of interfering with VLA‐4 activation, eventually decreasing soluble VCAM‐1 binding and reducing downstream ERK phosphorylation of blood‐residing CLL cells [127].

Trisomy 12 CLL cells are exceptionally high in both LFA‐1 and VLA‐4 expression (Fig. 3E), which cooperatively facilitate their entry into lymph nodes and contribute to exacerbated lymphadenopathy. The increased integrin expression, particularly of LFA‐1, in trisomy 12 CLL may be linked to hypomethylation of the *ITGB2* promoter [130]. Trisomy 12 CLL cells are more prone to use the lymph node axis of CCL21‐CCR7 activation of the integrins than the bone marrow axis of CXCL12‐CXCR4 induction [131], with a noted link to Notch signaling [132]. This relationship warrants further exploration regarding its implications for BCR signaling.

Although BCR signaling and integrin interactions involve cytoskeletal components, the precise mechanisms remain unclear. It is reasonable to assume that integrins may stabilize antigen recognition in CLL, thereby impacting BCR signaling by shaping actin‐dependent kinase assemblies. In the future, a better understanding of these mechanistic aspects might contribute to overcoming therapy resistance in CLL.

## Summarizing considerations

BCR signaling is a central driver of CLL pathogenesis, regulating cellular proliferation, survival, and interactions with the microenvironment while exhibiting clinically significant interpatient heterogeneity. In normal B cells, functional responses downstream of the BCR as a result of BCR signaling rely on the actin cytoskeleton. The interplay between BCR signaling and the actin cytoskeleton is complex and forms a deeply interwoven network including integrins. Through their bidirectional signaling, integrins influence adhesion, motility, and signaling pathways that intersect with BCR activity. BCR signaling can amplify integrin activation, forming a positive feedback loop that drives cytoskeletal remodeling and enhances interactions with the lymphoid microenvironment. Therefore, BCR–integrin interactions are bidirectional rather than unidirectional. In CLL, integrin expression and function are disturbed at several levels, with likely impacts on the BCR interplay. While normal B cells rely on LFA‐1 function, CLL pathophysiology is significantly determined by the VLA‐4 integrin, a CD49d/CD29 heterodimer. The extent of CD49d expression robustly allows categorization of CLL patients into subgroups with different prognosis and importantly, to define patient groups that have a higher propensity to progress under BTK inhibition. While there is a clear interaction of antigen‐dependent and BCR‐autonomous signaling with the integrin and back, the molecular players are not well defined yet and the bypass mechanisms of BTK in CD49d‐positive CLL patients remain to be resolved.

The therapeutic implications of the BCR signaling‐actin axis extend beyond CLL to other B cell malignancies that share similar signaling and cytoskeletal alterations. Given the heterogeneity of CLL cytoskeletal features, patient‐tailored therapeutic approaches are crucial and it might be useful to take into account the nature of BCR subsets and autonomous signaling, together with the CD49d status. CD49d expression as a biomarker, along with the BCR status assessment, may help to predict therapy responses, the potential of definite therapy regimes and side effects.

## Author contributions

AP and TNH wrote, revised, and edited the manuscript.
